# Analysis of Key Genes Involved in Potato Anthocyanin Biosynthesis Based on Genomics and Transcriptomics Data

**DOI:** 10.3389/fpls.2019.00603

**Published:** 2019-05-14

**Authors:** Nie Tengkun, Wang Dongdong, Ma Xiaohui, Chen Yue, Chen Qin

**Affiliations:** ^1^State Key Laboratory of Crop Stress Biology for Arid Areas, College of Agronomy, Northwest A&F University, Yangling, China; ^2^State Key Laboratory of Crop Stress Biology for Arid Areas, College of Food Science and Engineering, Northwest A&F University, Yangling, China

**Keywords:** anthocyanin, potato, multi-omics analysis, *stAN1*, *PAL*, R2R3-MYB

## Abstract

The accumulation of secondary metabolites, such as anthocyanins, in cells plays an important role in colored plants. The synthesis and accumulation of anthocyanins are regulated by multiple genes, of which the R2R3-MYB transcription factor gene family plays an important role. Based on the genomic data in the Potato Genome Sequencing Consortium database (PGSC) and the transcriptome data in the SRA, this study used potato as a model plant to comprehensively analyze the plant anthocyanin accumulation process. The results indicated that the most critical step in the synthesis of potato anthocyanins was the formation of *p*-coumaroyl-CoA to enter the flavonoid biosynthetic pathway. The up-regulated expression of the *CHS* gene and the down-regulated expression of *HCT* significantly promoted this process. At the same time, the anthocyanins in the potato were gradually synthesized during the process from leaf transport to tubers. New transcripts of *stAN1* and *PAL* were cloned and named *stAN1-like* and *PAL-like*, respectively, but the functions of these two new transcripts still need further study. In addition, the sequence characteristics of amino acids in the R2-MYB and R3-MYB domains of potato were preliminarily identified. The aims of this study are to identify the crucial major genes that affect anthocyanin biosynthesis through multi-omics joint analysis and to transform quantitative traits into quality traits, which provides a basis and reference for the regulation of plant anthocyanin biosynthesis. Simultaneously, this study provides the basis for improving the anthocyanin content in potato tubers and the cultivation of new potato varieties with high anthocyanin content.

## Introduction

It is well known that some plants are colorful, and there are many reasons why plants display multiple colors. For example, the pH of plant cytoplasmic substrates, the accumulation of secondary metabolites, such as anthocyanins, and environmental factors, such as light, all have an effect on plant color formation (Asen et al., 1972; Dai and Mumper, 2010; Xu X. et al., 2015). The accumulation of anthocyanins and other flavonoids in cells results in plants displaying colors other than green (Tanaka et al., 2008). Biosynthesis and metabolic pathways of anthocyanins in plants have been studied in depth, and many key genes have been cloned.

Among the many phenylalanine metabolic pathways, the pathway based on the biosynthesis process of phenylpropanoids is an important source of flavonoids in plants (Salvatierra et al., 2010). Phenylalanine is deaminated by phenylalanine ammonia lyase (PAL) to form *trans*-cinnamic acid; *trans*-cinnamic acid produces cinnamoyl-CoA under 4-coumarate-CoA ligase (4CL); then cinnamoyl-CoA is catalyzed by *trans*-cinnamate 4-monooxygenase (C4H) to form *p*-coumaroyl-CoA; finally *p*-coumaroyl-CoA is involved in the biosynthesis of flavonoids (Vogt, 2010). *p*-coumaroyl-CoA, through chalcone synthase (CHS), shikimate *O*-hydroxycinnamoyltransferase (HCT), chalcone isomerase (CHI), flavonoid 3′, 5′-hydroxylase (F3′5′H), flavonoid 3′-monooxygenase (F3′H), naringenin 3-dioxygenase (F3H), dihydroflavonol 4-reductase (DFR), anthocyanidin synthase (ANS) and other enzymes, catalyzes the final formation of pelargonidin, cyanidin and delphinidin, involved in anthocyanin biosynthesis (Martens et al., 2010; Tanaka et al., 2010). Anthocyanin mainly accumulates in plant cell vacuoles in the form of glycosides (Pietrini et al., 2002).

The MYB-bHLH-WD40 transcription factor complex (MBW) is a regulator that has been thoroughly studied and has an important regulatory effect on the synthesis of flavonoids such as anthocyanins (Jaakola, 2013). The main transcription factor involved in the regulation of anthocyanin synthesis in the MYB gene family is the R2R3-MYB transcription factor (Stracke et al., 2007). A study of the *Arabidopsis* MBW complex TT2-TT8-TTG1 showed that the target gene of the complex might be mainly determined by a R2R3-MYB transcription factor-encoded protein (Xu W. et al., 2015). The bHLH proteins involved in the MBW complex have some common features and most belong to the IIIF subfamily (Zimmermann et al., 2004). The *Arabidopsis thaliana TT8* gene belongs to the bHLH gene family, which can regulate the synthesis of flavonoids by feedback regulation (Baudry et al., 2006). Studies have indicated that the WD40 protein does not participate in the recognition of gene promoters or regulate the expression of target genes; its effect is to link the two other protein subunits in the MBW complex (Hichri et al., 2011). In the synthesis of flavonoids, for some specific genes, MYB transcription factors can activate the corresponding gene transcription directly without binding to bHLH transcription factors (Jaakola, 2013). Thus, it is important that the R2R3-MYB transcription factor plays a role in the synthesis of flavonoids.

Anthocyanin is an important component of polyphenolic antioxidant active substances, and such compounds are easily absorbed and utilized by the human digestive system (Fernandes et al., 2014). Anthocyanins have a special chemical structure, which allows them to exert a variety of physiological and biochemical functions in mammals such as humans (Stintzing and Carle, 2004). On the one hand, anthocyanins have the effect of scavenging free radicals in living organisms and improving the antioxidant capacity of organisms themselves (Miguel, 2011); on the other hand, anthocyanins have many important pharmacological effects, for example, anthocyanins have significant effects in preventing many major human-related diseases, such as cardiovascular and cerebrovascular diseases, diabetes and its complications, cancer, and so on (Scalbert et al., 2005). Because of the above characteristics, anthocyanins are gradually being valued by chemists and pharmacologists. Potato is an important plant food for humans to obtain antioxidant active substances such as ascorbic acid and polyphenols (Lobo et al., 2010). Nutrients such as anthocyanins accumulate in colored potato tubers. In addition, it is considered that the anthocyanin content of potato with red or purple tubers is significantly higher than that of common potato with white or yellow tubers (Brown et al., 2005; Lachman and Hamouz, 2005). Since anthocyanins have favorable biological functions for humans, the key genes controlling the synthesis and accumulation of potato anthocyanins can be studied, and then the accumulation of anthocyanins in potato tubers can be regulated. This study attempted to control the content of anthocyanins in potato tubers, making it easier for humans to take antioxidant active substances such as anthocyanins, thereby preventing a variety of diseases and making humans healthier.

Potato is a good model plant for studying the formation of plant color by studying the process of anthocyanin biosynthesis. Firstly, potato plants reproduce mainly through asexual reproduction, and the genetic composition is stable. Secondly, different potato varieties have different colors, and for a single potato, the whole plant is consistent in color. In addition, mature potato plants have a large biomass, which is convenient for the determination of various secondary metabolites. Numerous key genes regulating anthocyanin synthesis have been cloned, but it is unclear which of these key genes is the most important. At the same time, whether there are other gene regulatory pathways controlling anthocyanin accumulation in plants is also worthy of further study.

In this experiment, we analyzed the R2R3-MYB transcription factor gene family, which plays a major role in the anthocyanin synthesis process, based on the genomic data of existing diploid potato (*Solanum phureja* DM1-3). Then, potato transcriptomics data from the NCBI Sequence Read Archive (SRA) database were used to determine which key genes were enriched in anthocyanin synthesis. Finally, based on the above analysis results, we aimed to identify the most critical genes involved in the regulation of anthocyanin biosynthesis and to explore new genes that may be involved in the regulation of anthocyanin synthesis.

## Materials and Methods

### Identification of the R2R3-MYB Subfamily Genes in Potato Proteome Data

We downloaded proteomic data PGSC_DM_v3.4_pep.fasta (Amino acid sequences corresponding to all gene coding sequences) from the potato group database PGSC^1^. The identification of R2R3-MYB subfamily genes used *stAN2* as a reference sequence (Jung et al., 2009); local Blast analysis was performed using blast-2.6.0+ software, and the e-value was set to e-5. After removal of short sequences of amino acids with a length less than 100 and repeated sequences, the SMART^2^ database was submitted for retrieval. MEME 4.11.4^3^ was used to determine the conserved domain boundaries of the MYB-R2 and MYB-R3 domains in potato. Only the amino acid sequences having both the MYB-R2 and MYB-R3 domains were retained for subsequent analysis.

### Construction of the Phylogenetic Tree of the Potato R2R3-MYB Gene and Collinear Analysis

Using MEGA7^4^ software, an unrooted tree was constructed using the minimal evolution method, and the phylogenetic tree was tested using Bootstrap = 1000. The potato genome collinearity analysis was performed based on the PGSC_DM_v3.4_cds.fasta application MCScanX^5^, and circos-0.69^6^ was used to visualize the results of the potato genome collinearity analysis.

### Transcriptional Data of Potato Color Changes Were Analyzed

The potato transcriptome data were downloaded from the SRA database^7^ the downloaded data format was transformed by the SRA-Toolkit^8^, and then the downloaded data were regrouped. According to the color of the potato stem and tuber used in sequencing, they were reclassified into a colored group and colorless group. The regrouped colored group contained 21 biological replicates; the regrouped colorless group contained 36 biological replicates. The colorless group was the control group, and the data and grouping information are shown in Supplementary Table S5 (Hannapel et al., 2013; Liu et al., 2015; Gálvez et al., 2016; Pham et al., 2017). In this experiment, the NGSQC Toolkit (Patel and Jain, 2012) was used to filter the reads; Trimmomatic^9^ was used to remove the linkers used for sequencing; and the PCR repeats generated during the sequencing process were eliminated by FastUniq^10^. Using the doubled monoploid *S. tuberosum* Group Phureja clone DM1-3 (DM) as the reference genome (Xu et al., 2011), TopHat and Cufflinks were used to splice the transcriptome data and obtain differentially expressed genes (Trapnell et al., 2012). Finally, InterProScan-5.29-68.0^11^ and KOBAS 3.0^12^ were used for preliminary annotations of the differentially expressed genes.

### GO Annotation and KEGG Enrichment Analysis Based on Genomic and Transcriptome Analysis Results

Comprehensive genomic and transcriptome analysis results were analyzed by GO annotation and KEGG enrichment using AnnotationDbi^13^, AnnotationHub^14^ and clusterProfiler^15^. Only GO annotations and KEGG enrichment analysis results with *p*-value < 0.05 were retained. The GOplot^16^ was applied to visualize the results of GO annotation. The KEGG analysis results were confirmed by the KEGG online database^17^.

### Semi-Quantitative RT-PCR to Detect Gene Expression

Semi-quantitative RT-PCR was used to verify the expression of the key genes obtained from the above studies. We applied the potato variety Shepody and the colored potato material, Yellow Meigui 1, Red Meigui 3, Purple Meigui 2, which were bred in our laboratory. The color performance of each potato material is shown in Figure 5C. In this experiment, total RNA of roots, stems, leaves, and tubers of potato seedlings was extracted by TRNzol. After reverse transcription, semi-quantitative RT-PCR was carried out with *EF-1α* as the reference gene. The semi-quantitative RT-PCR experiment of each plant tissue was performed with 5 biological replicates. The primers used in the above experiments are shown in Supplementary Table S6. Finally, ImageJ^18^ was used to measure the agarose gel gray value and perform statistical analysis.

### Application of Tobacco Leaves for Subcellular Localization

The *stAN1-like*-GFP vector and the *PAL-like*-GFP vector were constructed and transformed into *Agrobacterium* strain LBA4404 by the freeze-thaw method. The transformed *Agrobacterium* was cultured at 28°C with shaking until the OD_600_ = 0.6 – 0.8, and the cells were centrifuged. We used a suspension (MES = 10 mmol/L; MgCl_2_ = 10 mmol/L; acetosyringone = 0.3 mmol/L; pH = 5.8) to resuspend the cells. The resuspended cells were allowed to stand at room temperature for 2 h, and the resuspended bacteria were injected into the tobacco leaves using a disposable syringe. Under the condition of maintaining the humidity, green fluorescence was observed by laser scanning confocal microscopy (LSCM) after 48 h of tobacco leaf injection. The injected tobacco leaves were treated with a 0.25 g/ml sucrose solution, and the plasmolysis was observed by LSCM (Supplementary Figure S1). The GFP excitation wavelength was 488 nm, and the chloroplast autofluorescence excitation wavelength was 633 nm.

## Results

### Identification of Genes Containing Only the R2 and R3 Domains in the Potato MYB Family

**FIGURE 1:**
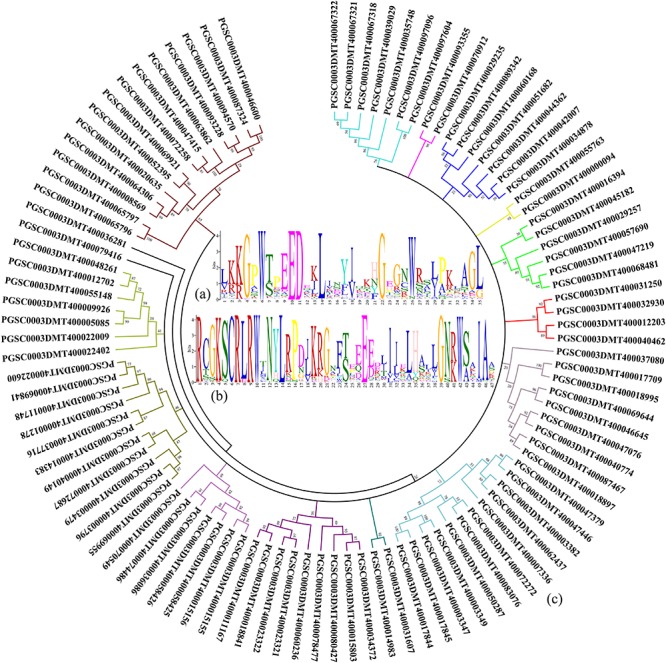
Genes with the R2R3-MYB domain are identified in potato. **(A)** Characteristics of the R2 domain in the potato MYB gene family. **(B)** Amino acids are contained in the R3 domain of the potato MYB gene family. **(C)** Homology analysis of protein sequences are translated by genes with the R2R3-MYB domain in potato.

In the potato genome data, a total of 101 genes with the R2R3-MYB domain were found by a literature search and sequence alignment (Jung et al., 2009; Zhao et al., 2013; Liu et al., 2016). By comparing the protein sequences found using the above genes, the common features of the functional structure of the potato R2R3-MYB gene family were obtained. The results of the alignment of the R2 domain, which contains a total of 35 amino acids, are shown in Figure 1A. Analysis of the R3 domain revealed a total of 47 amino acids in its domain (Figure 1B). In the R2 and R3 domains, the conserved amino acids in order from the N-Terminal to the C-Terminal are glycine (G), tryptophan (W), glutamic acid (E), glycine (G), and tryptophan (W). Therefore, the G-W-E-G-W structure may have an important function in the process of binding the MYB transcription factor to the target promoter.

A phylogenetic tree was constructed using the amino acid sequence corresponding to the gene with the R2R3-MYB domain found in potato. As shown in Figure 1C, the population of genes could be initially divided into 16 subpopulations based on the amino acid homology alignment. Amino acid homology analysis provided a reference for finding genes with the R2R3-MYB domain in the potato genome associated with anthocyanin accumulation.

### Collinearity Analysis of Potato R2R3-MYB Genes

**FIGURE 2:**
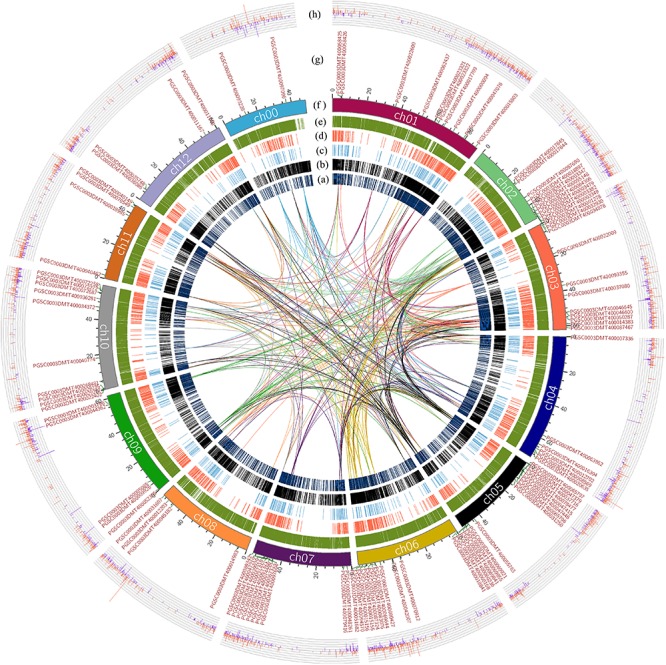
Collinearity analysis of the R2R3-MYB domain genes in the potato genome, and gene expression differences based on transcriptome analysis. The lines indicate the collinearity of R2R3-MYB genes in the potato genome, and the line color is the same as the chromosome color corresponding to R2R3-MYB genes. **(A)** The distribution of the singleton-type genes in the potato genome. **(B)** Distribution of dispersed duplication type genes in the potato genome. **(C)** Distribution of the proximal type genes. **(D)** Distribution of the tandem type genes. **(E)** Distribution of WGD/segmental type genes in potato. **(F)** The length of each chromosome of the potato is expressed in units of Mb, where in the ch00 chromosome, are unanchored sequences based on the sequencing result of the potato DM genome. **(G)** The distribution of genes with the R2R3-MYB domain on each chromosome of potato. **(H)** The difference in the expression of each gene obtained by transcriptome analysis showed that the data were differential genes of |log_2_FC| ≥ 1. The red color indicates that the genes were up-regulated in the colored group; the blue color indicates that the genes were down-regulated in the colored group.

The whole genome of potato was analyzed by collinearity analysis. The results showed that the potato genes were divided into five types: no repeat genes (singleton); modes other than segmental, tandem and proximal (dispersed duplication); nearby chromosomal region but not adjacent (proximal); consecutive repeat (tandem); and collinear genes in collinear blocks (WGD/segmental). Among them, the proximal type had a minimum of 1441 genes; the WGD/segmental type had a maximum of 21372 genes. The remaining types were 4797 genes for the singleton type, 7408 genes for the dispersed duplication type, and 4011 genes for the tandem type gene (Figures 2A–E). There were 25,383 collinear genes and tandem replication genes in the potato genome, accounting for 65.04% of the total number of potato genes. It could be seen that most genes had multiple copies in the potato genome, and there was a high number of genes with similar sequence characteristics or functions.

R2R3-MYB genes were present on each chromosome of potato. The R2R3-MYB genes were most abundantly distributed on the ch05 chromosome, with a total of 14 R2R3-MYB genes on this chromosome. Furthermore, the R2R3-MYB genes were also extensively distributed on the ch01, ch02, ch03, ch06, ch07, and ch10 chromosomes (Figure 2G). The distributions of the collinear genes and the tandem genes in the potato genome were relatively uniform on each chromosome, but there were fewer in the 41–46 Mb region of ch00 and the 1–10 Mb region of ch02. The lines in Figure 2 indicated the collinear relationship between R2R3-MYB genes in the potato genome and between the R2R3-MYB genes and other genes in potato. Based on the above results, a total of 31 other genes were found in the potato genome, which were collinear with the members of the R2R3-MYB gene family identified above (Supplementary Table S1). Genes that were collinear with the R2R3-MYB gene family members could also be used as key candidate genes for the regulation of potato anthocyanin synthesis.

### Transcriptome Analysis Results

Based on the re-grouping transcriptome sequencing data, a total of 12,913 genes with different expression levels were found, of which 420 (*p* ≤ 0.05) were significantly different in terms of expression (Supplementary Table S2). There were 11030 genes with different expression levels |log2FC| ≥ 1; the colored group up-regulated genes accounted for 58.52%, and the colored group down-regulated genes accounted for 41.48% (Figure 2H). Compared with the colorless group, the number of up-regulated genes in the colored group was significantly higher. This indicated that the change in plant color and the accumulation of anthocyanins were achieved by the simultaneous up-regulation of multiple genes.

### GO Enrichment and KEGG Path Analysis

**FIGURE 3:**
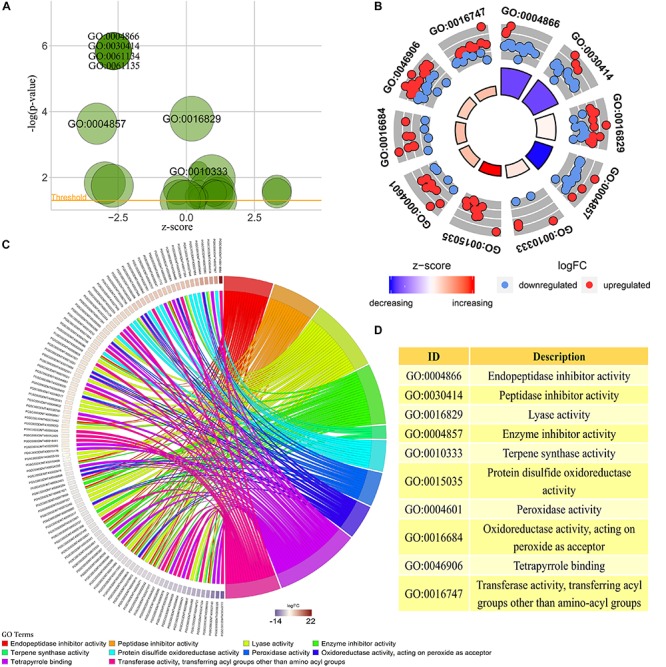
GO enrichment analysis of transcriptome sequencing results. **(A)** All GO enrichment results with *p*-value < 0.05, and the results noted in the figure are GO enrichment results with *p*-value < 0.01. **(B)** GO annotation pathways associated with anthocyanin biosynthesis based on GO enrichment results with *p*-value < 0.05. **(C)** Differentially expressed potato genes based on the results of the GO pathway associated with anthocyanin biosynthesis in the above analysis. The higher the logFC, the higher the expression of genes in the potato colored group, and vice versa. **(D)** Detailed description of key GO enrichment pathways.

GO enrichment analysis was performed on transcriptome data using interproscan and clusterProfiler software (Yu et al., 2012; Jones et al., 2014). A total of 23 valid GO annotation terms (*p*-value < 0.05) were enriched, of which there were 7 annotation results with *p*-value < 0.01 (Figure 3A). The content of anthocyanins or polyphenols in plants has a close positive correlation with the antioxidant activity of plants (Velioglu et al., 1998). Among the 23 GO analysis results, 10 were significantly associated with plant color changes or plant antioxidant activity. Among them, the GO:0015035, GO:0004601, GO:0016684, GO:0046906, GO:0016747, GO:0010333, and GO:0016829 pathways were enhanced in the colored potato group, whereas the GO:0004866, GO:0030414, and GO:0004857 pathway were weakened in the colored potato group (Figures 3B,D).

It could be seen that the antioxidant activity of potato in the colored group was stronger than that in the colorless group, and the acyltransferase activity of potato in the colored group was also higher than that in the colorless group. This indicated that the high expression of some antioxidant genes and acyltransferase genes contributes to the accumulation of substances such as anthocyanins in plants. At the same time, it also showed that colored potato had higher antioxidant activity, and the antioxidant activity was improved by the simultaneous up-regulation of multiple key genes. A total of 104 differentially expressed genes were enriched in 10 significantly GO pathways, and these gene expressions may play an important role in the accumulation of potato anthocyanins (Figure 3C). Therefore, the above genes can be used as key candidate genes for further study of the synthesis of plant flavonoids and changes in plant antioxidant activity.

**FIGURE 4:**
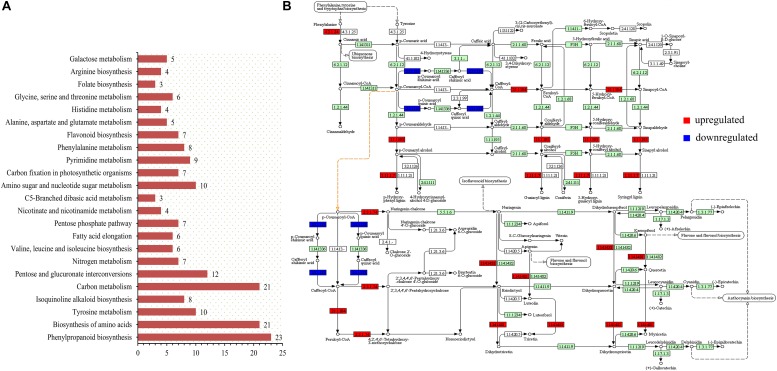
KEGG pathway analysis was performed on transcriptome sequencing data. **(A)** Overview of KEGG analysis results (*p*-value < 0.05). **(B)** Genes with obvious changes in the expression of phenylpropanoid and flavonoid biosynthesis pathways in the colored potato group (|logFC| > 1).

The transcriptome data were enriched by KEGG analysis to obtain 23 metabolic pathways (*p*-value < 0.05), including two pathways closely related to anthocyanin synthesis and accumulation (Figure 4A). These two pathways were sot00940 (phenylpropanoid biosynthesis) and sot00941 (flavonoid biosynthesis). The biological processes related to the accumulation of anthocyanins were sorted, and the up- and down-regulated expression changes of the potato genes in the colored group are shown in Figure 4B. The role of PAL (4.3.1.24) in phenylpropanoid biosynthesis is very important, but this study found that its up-regulated expression in colored potatoes was not obvious. However, the enhancement of the enzyme activity of caffeoyl-CoA *O*-methyltransferase (2.1.1.104), cinnamyl-alcohol dehydrogenase (1.1.1.195), and peroxidase (1.11.1.7) in the colored group promoted the formation of various phenolic substances, represented by lignin, and also promoted the transformation of cinnamoyl-CoA into *p*-coumaroyl-CoA.

*P*-cinnamoyl-CoA is a key precursor of synthetic anthocyanins, and its increased content contributes to the accumulation of potato anthocyanins (Besseau et al., 2007). The up-regulated expression of PGSC0003DMT400022254 and PGSC0003DMT400022255 genes increased the content of the CHS (2.3.1.74) enzyme and promoted the accumulation of downstream products, which is of great significance in the whole process of anthocyanin accumulation. During the whole process of anthocyanin synthesis, the expression level of the PGSC0003DMT400018861 gene was significantly decreased, resulting in a decrease in the HCT (2.3.1.133) content. This could effectively reduce the loss of *p*-coumaroyl-CoA to caffeic acid metabolism and promote *p*-coumaroyl-CoA in the flavonoid synthesis pathway, which also had a positive significance for the accumulation of anthocyanins. In addition, the up-regulation of F3′5′H (1.14.14.81) could effectively counteract the effect of HCT (2.3.1.133) down-regulated expression on the anthocyanin composition type. This resulted in the contents of the delphinidin, pelargonidin, and cyanidin classes remaining relatively balanced.

### Semi-Quantitative RT-PCR to Verify the Expression of Related Genes

The members of the potato R2R3-MYB gene family were preliminarily identified by sequence alignment and construction of a phylogenetic tree, and the characteristics of R2 and R3 domains in potato were determined. Based on the collinearity analysis of the R2R3-MYB gene family, the R2R3-MYB gene family members were further enriched. A total of 104 potato R2R3-MYB gene family members were identified by combining phylogenetic analysis and collinearity analysis. Combined with the results of transcriptome analysis, the differentially expressed genes were searched for among the 104 R2R3-MYB members, and the most differentially expressed genes may be related to the synthesis of anthocyanins and changes in potato color.

**FIGURE 5:**
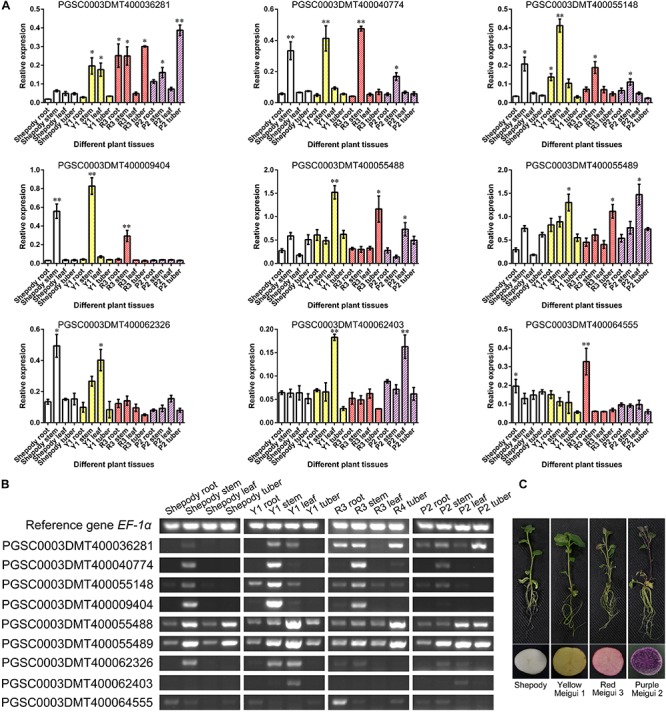
Verification of key gene expression. **(A)** Relative expression levels of key genes obtained by semi-quantitative RT-PCR. The semi-quantitative RT-PCR experiment of each plant tissue was performed on 5 biological replicates. **(B)** The results of agarose gel electrophoresis corresponding to semi quantitative RT-PCR tests. **(C)** Potato materials used in this experiment. Y1, Yellow Meigui 1; R3, Red Meigui 3; P2, Purple Meigui 2. *Significant difference (*p*-value < 0.05); **highly significant difference (*p*-value < 0.01).

Based on a comprehensive comparison of genomic and transcriptome analysis results (Supplementary Tables S3, S4), a total of 9 genes were further confirmed. The results of transcriptome analysis were verified by semi-quantitative RT-PCR using colored potatoes as material (Figure 5C). The expression of 7 genes was the same as that of transcriptome analysis, and the expression of PGSC0003DMT400062326 and PGSC0003DMT400062403 was opposite to that of transcriptome analysis (Figures 5A,B). PGSC0003DMT400040774, PGSC0003DMT400055148, and PGSC0003DMT400009404 were mainly expressed in the potato stem. The expression level of PGSC0003DMT400064555 in various tissues of colored potatoes was generally lower than that of the control Shepody, but higher in the root of Red Meigui 3. PGSC0003DMT400055488 (*PAL-like*) was expressed in leaves and tubers of colored potatoes, but the expression did not increase with the deepening of potato color. The expression levels of PGSC0003DMT400036281 (*stAN1-like*) and PGSC0003DMT400055489 (*PAL*) increased as the color of the potato deepened. *Solanum tuberosum* anthocyanin 1 like (*stAN1-like*) was mainly expressed in the roots, stems and tubers of potato; its expression in Red Meigui 3 and Purple Meigui 2 potato tubers was significantly increased. The expression of phenylalanine ammonia-lyase (*PAL*) was mainly concentrated in the leaves of colored potatoes, but the expression level in the leaves of the control variety Shepody was significantly reduced.

### Subcellular Localization of *stAN1-Like* and *PAL-Lik*e

**FIGURE 6:**
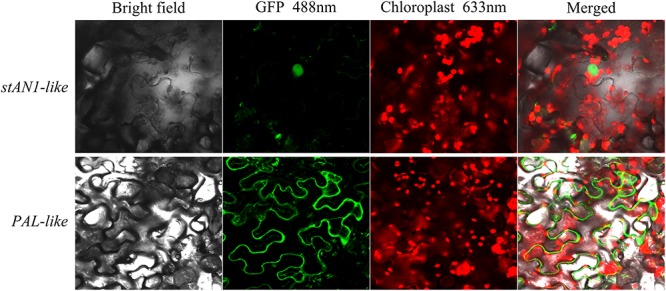
Subcellular localization of *stAN1-like* and *PAL-like* using tobacco leaves.

The total RNA of leaves was extracted from the Red Meigui 3 potato, and the new transcripts *stAN1-like* and *PAL-like* of *stAN1* and *PAL* genes were cloned by RT-PCR. The length of the CDS sequence of *stAN1-like* is 798 bp, which indicates that the resulting protein peptide chain contains 265 amino acids. The length of the CDS sequence of *PAL-like* is 2169 bp, and 722 amino acids are included in the protein peptide chain. The subcellular localization results of *stAN1-like* (PGSC0003DMT400036281) and *PAL-like* (PGSC0003DMT400055488) genes are shown in Figure 6. It could be seen that the proteins produced by the *stAN1-like* guide were mainly concentrated in the nucleus. This suggested that *stAN1-like* might have the function of initiating downstream gene expression. The protein translated by *PAL-like* was concentrated on the cell membrane (Supplementary Figure S1), which is consistent with its function as a functional protein to promote the conversion of phenylalanine to anthocyanin-producing precursor phenylpropanoids. Some of the phenylpropanoids are further metabolized to form lignins involved in cell wall synthesis (Zhou et al., 2009).

## Discussion

### Distribution of Potato R2R3-MYB Transcription Factor on Chromosomes

The R2R3-MYB transcription factor genes have important functions in the process of anthocyanin biosynthesis (Feller et al., 2011). Their primary function in the MBW transcriptional complex is binding to a gene (Xu W. et al., 2015). In this study, 101 R2R3-MYB family genes were found in the potato genome, which were distributed on all of the chromosomes of potato. This indicates that R2R3-MYB transcription factor genes have important biological functions in potato. R2R3-MYB family genes not only participate in the synthesis and regulation of flavonoids, such as anthocyanins, but also participate in many physiological and biochemical processes, such as floral induction, photoperiod response, and plant drought resistance, and so on (Albert et al., 2011; Yang et al., 2012; Zhang et al., 2012; Liu et al., 2013). In addition, in other crops, such as *Arabidopsis* and *Oryza sativa*, the R2R3-MYB transcription factors were also found to be distributed on all of the chromosomes (Katiyar et al., 2012). This further demonstrates that the functions of the R2R3-MYB transcription factors are important for plants.

### The Function of New Transcripts of *stAN1* and *PA*L

The new transcripts of *stAN1* and *PAL* in this experimental clone were from our own laboratory material Red Meigui 3. The new transcripts were named *stAN1-like* and *PAL-like*, respectively. The cloned *stAN1-like* amino acid sequence differs from *stAN1* (Supplementary Figure S2), which has been reported to regulate potato color (Zhang et al., 2009; Liu et al., 2016). The stAN1-like transcript has 21 bases more than the 5′ end of the stAN1 reference transcript. By comparing the stAN1-like transcript with the stAN1 reference gene sequence, it was found that the 21 bases were completely identical to the stAN1 reference gene sequence. It can be clarified that the production of stAN1-like transcripts is caused by the changes of transcription initiation sites or splicing sites of the pre-mRNA. Therefore, it is necessary to further study the role of *stAN1-like* in potato anthocyanins synthesis and plant color change. The *PAL* gene also plays an important role in the accumulation of potato anthocyanins (Zhang and Liu, 2015), but *PAL-like* is different from the typical *PAL* gene (Supplementary Figure S3). Therefore, it is impossible to rule out the possibility that proteins produced by *PAL-like* guidance have other functions. The function of *PAL-like* needs further research through molecular biological methods.

### Biosynthesis and Accumulation Process of Anthocyanins

The R2R3-MYB transcription factor mainly regulates the transcription of downstream genes controlling anthocyanin synthesis, such as DFR (Nesi et al., 2001). The results of comprehensive transcriptome analysis showed that the upstream genes controlling the synthesis of anthocyanin precursors represented by *PAL* (PGSC0003DMT400055489) were mainly expressed in leaves. However, the R2R3-MYB transcription factor genes represented by *stAN1-like* (PGSC0003DMT400036281) were mainly concentrated in stems and tubers. This indicates that there is a transport process during the synthesis and accumulation of anthocyanins throughout the potato. Anthocyanin precursors such as phenylalanine and tyrosine accumulate in leaves; then the intermediate products are gradually catalyzed to form the final product (anthocyanins) in the process of transport to the tubers; the final end product accumulates in the tuber in the form of anthocyanins. The whole process is synthesized while transporting, rather than directly accumulating the final product of anthocyanin biosynthesis in the leaves and then transferring to the tubers.

Analysis of transcriptomic data revealed that the role of *PAL* gene in the overall anthocyanin biosynthesis process is not critical. In anthocyanin biosynthesis, the metabolic step that really plays a pivotal role should be the following process: The anthocyanin synthesis precursor *p*-cinnamoyl-CoA is transformed into naringenin chalcone as much as possible, thereby entering the subsequent synthesis process of anthocyanins, so that *p*-cinnamoyl-CoA enters the synthesis pathway of lignin as little as possible. In colored potatoes, the expression of *CHS* was up-regulated, and the down-regulated expression of *HCT* effectively realized this process. Therefore, the up-regulation of *CHS* and the down-regulation of *HCT* should be the most critical link to promote plant anthocyanin synthesis and increase the plant anthocyanin content. In addition, the high expression of the *F3′5′H* gene effectively offsets the effect of the down-regulated expression of *HCT* on the anthocyanin composition type, so that the composition of each type of anthocyanin can remain relatively balanced.

### Application of Multi-Omics Joint Analysis in Experiments

With the development of bioinformatics and the accumulation of experimental data in the field of plant life sciences, it has become possible for multi-omics to jointly analyze a certain life phenomenon (Zhang et al., 2010; Lakshmanan et al., 2015). The transcriptomics data used in this paper were different from the traditional RNA-seq data. This experiment combined multiple RNA-seq results for comprehensive analysis. Potato could be used as a good model plant to study the process of anthocyanin synthesis and accumulation. However, due to the lack of research on potato gene function, it is difficult to perform transcriptome analysis and annotation, especially for transcription factor-related genes. At the same time, potato proteomics and metabolomics experimental data are still insufficient, and the analytical methods are limited, which make the relevant life phenomena unable to be fully analyzed. Future scientific research needs to further complement data on potato-related proteomics, metabolomics, and phenomics. With the advancement of life sciences, the above problems will surely be gradually solved.

The anthocyanin metabolism and synthesis process is a typical quantitative trait, and the synthesis process is controlled by multiple genes. In this experiment, the genomic and transcriptome analysis indicated that the most important step in the anthocyanin synthesis process was to transfer *p*-cinnamoyl-CoA into the flavonoid biosynthesis process instead of further metabolism-producing lignin species. Up-regulation of *CHS* and down-regulation of *HCT* played a central role in anthocyanin biosynthesis. Through this analysis, we strived to find the major genes that regulate quantitative traits and convert quantitative traits into quality traits. At the same time, it was preliminarily found that anthocyanins synthesized precursor substances in leaves that were then gradually transformed during transport, and finally, end products (anthocyanins) accumulated in potato tubers. After a comprehensive analysis, two new transcripts with research potential were found, namely, *stAN1-like* and *PAL-like*, and their functions were preliminarily studied. However, the specific functions of these two transcripts still require the construction of transgenic plants for further research and validation. This study provides a reference for the comprehensive analysis and application of multiple transcriptomics data in the context of big data. At the same time, it also provides a reference for the application of R programming language in GO and KEGG analysis of non-model plants. Finally, the results of this study provide a solid theoretical basis for increasing the anthocyanin content in potato tubers, cultivating new potato varieties with high anthocyanin content and regulating plant color.

## Author Contributions

NT completed the main content of this manuscript. WD and MX made language retouching of this manuscript. CQ and CY provided guidance for the experiments.

## Conflict of Interest Statement

The authors declare that the research was conducted in the absence of any commercial or financial relationships that could be construed as a potential conflict of interest.

## Footnotes

1 http://solanaceae.plantbiology.msu.edu/pgsc_download.shtml

2 http://smart.embl-heidelberg.de/

3 http://meme-suite.org/index.html

4 http://megasoftware.net/

5 http://chibba.pgml.uga.edu/mcscan2/

6 http://circos.ca/software/download/circos/

7 https://www.ncbi.nlm.nih.gov/sra

8 https://trace.ncbi.nlm.nih.gov/Traces/sra/sra.cgi?view=software

9 http://www.usadellab.org/cms/index.php?page=trimmomatic

10 https://sourceforge.net/projects/fastuniq/files/

11 http://www.ebi.ac.uk/interpro/download.html

12 http://kobas.cbi.pku.edu.cn/

13 http://www.bioconductor.org/packages/devel/bioc/html/AnnotationDbi.html

14 http://www.bioconductor.org/packages/release/bioc/html/AnnotationHub.html

15 http://www.bioconductor.org/packages/release/bioc/html/clusterProfiler.html

16 http://wencke.github.io/

17 https://www.genome.jp/kegg/pathway.html

18 https://imagej.nih.gov/ij/

## Supplementary Material

FIGURE S1 *PAL-like* transient expression of tobacco leaves with plasmolysis.

FIGURE S2 The amino acid sequence of *stAN1-like* was aligned with the reference sequence.

FIGURE S3 The amino acid sequence of *PAL-like* was aligned with the reference sequence.

TABLE S1 Other candidate genes found by collinear analysis.

TABLE S2 Genes with significantly different expression in the transcriptome.

TABLE S3 Genes with differential expression in the potato MYB-R2R3 gene family.

TABLE S4 The results of GO and KEGG analysis related to anthocyanin biosynthesis and accumulation based on transcriptome data.

TABLE S5 RNA sequencing data regrouping.

TABLE S6 Primers used in this experiment.
