# Supplementary figures and images for: Analysis of Key Genes Involved in Potato Anthocyanin Biosynthesis Based on Genomics and Transcriptomics Data
Source: Front Plant Sci. 2019 May 14;10:603. doi: 10.3389/fpls.2019.00603 (PMC6527903; doi:10.3389/fpls.2019.00603)

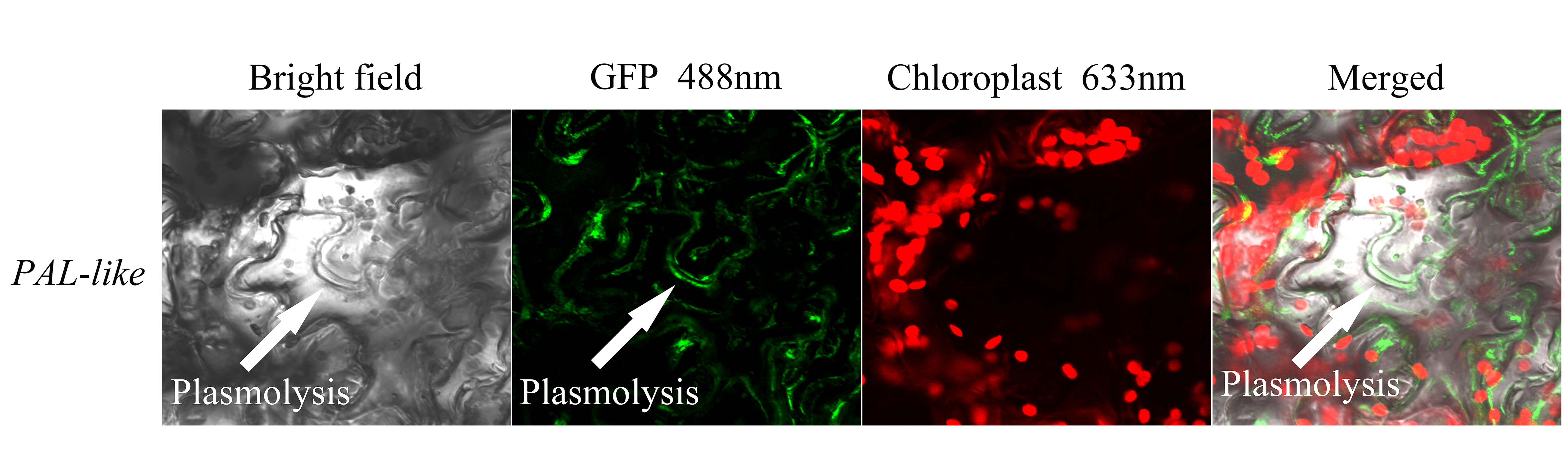

Supplement: FIGURE S1 — PAL-like transient expression of tobacco leaves with plasmolysis. [file Image_1.TIF]

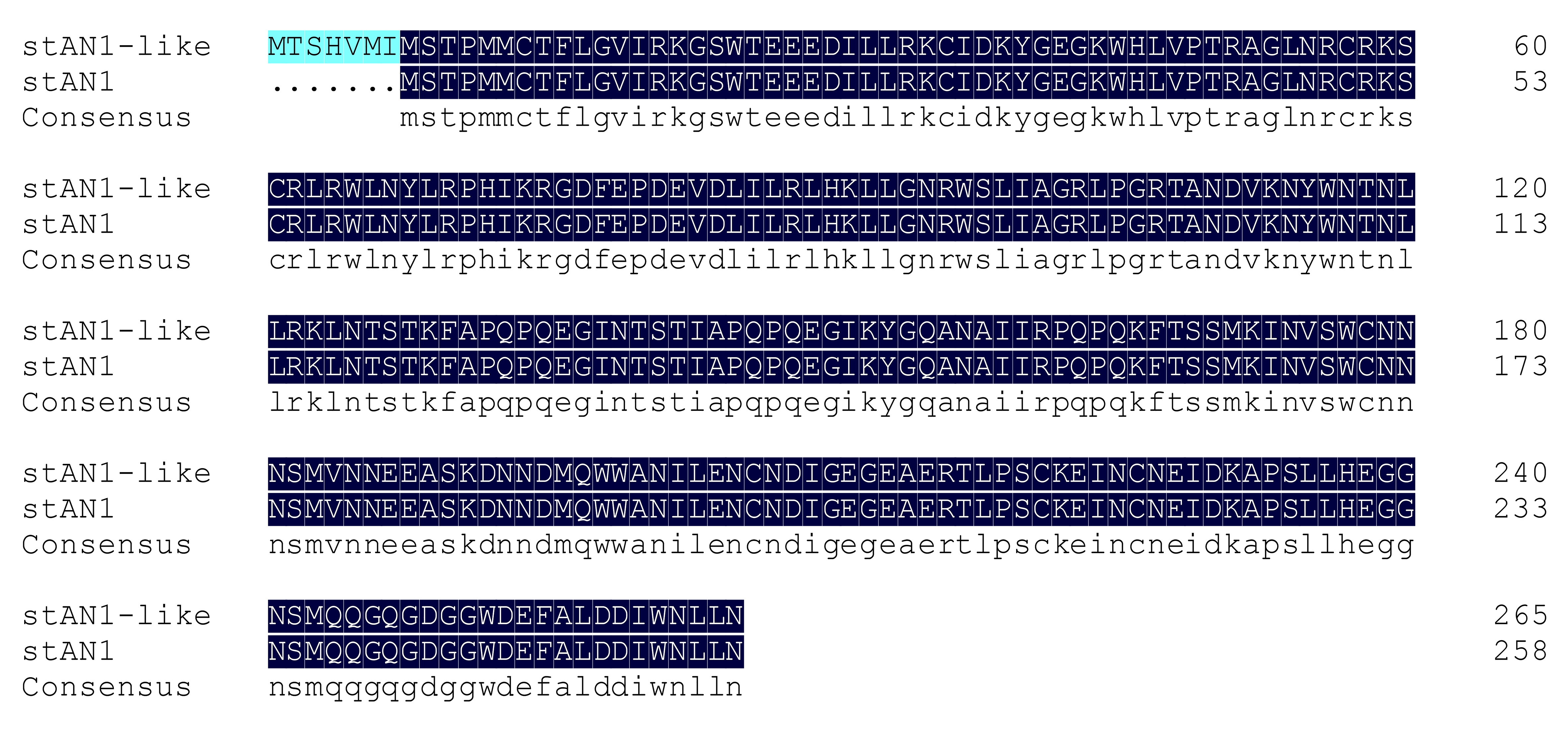

Supplement: FIGURE S2 — The amino acid sequence of stAN1-like was aligned with the reference sequence. [file Image_2.TIF]

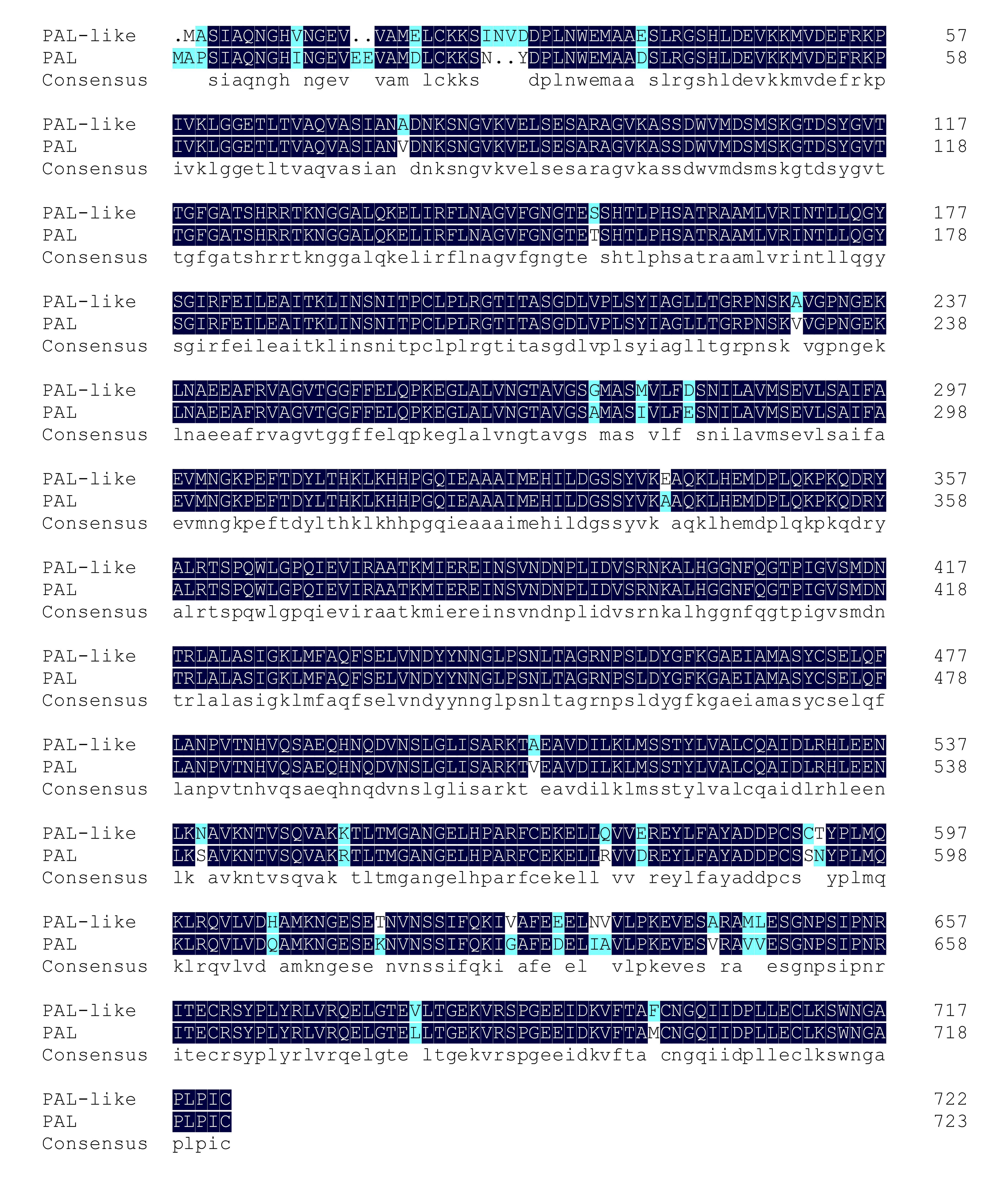

Supplement: FIGURE S3 — The amino acid sequence of PAL-like was aligned with the reference sequence. [file Image_3.TIF]
